# Characterizing collective physical distancing in the U.S. during the first nine months of the COVID-19 pandemic

**DOI:** 10.1371/journal.pdig.0000430

**Published:** 2024-02-06

**Authors:** Brennan Klein, Timothy LaRock, Stefan McCabe, Leo Torres, Lisa Friedland, Maciej Kos, Filippo Privitera, Brennan Lake, Moritz U. G. Kraemer, John S. Brownstein, Richard Gonzalez, David Lazer, Tina Eliassi-Rad, Samuel V. Scarpino, Alessandro Vespignani, Matteo Chinazzi

**Affiliations:** 1 Network Science Institute, Northeastern University, Boston, Massachusetts, United States of America; 2 Cuebiq Inc., New York, New York, United States of America; 3 University of Oxford, Oxford, United Kingdom; 4 Boston Children’s Hospital, Boston, Massachusetts, United States of America; 5 Harvard Medical School, Boston, Massachusetts, United States of America; 6 University of Michigan, Ann Arbor, Michigan, United States of America; 7 Vermont Complex Systems Center, University of Vermont, Burlington, Vermont, United States of America; 8 Santa Fe Institute, Santa Fe, New Mexico, United States of America; 9 ISI Foundation, Turin, Italy; 10 The Roux Institute, Northeastern University, Portland, Maine, United States of America; Tsinghua University, CHINA

## Abstract

The COVID-19 pandemic offers an unprecedented natural experiment providing insights into the emergence of collective behavioral changes of both exogenous (government mandated) and endogenous (spontaneous reaction to infection risks) origin. Here, we characterize collective physical distancing—mobility reductions, minimization of contacts, shortening of contact duration—in response to the COVID-19 pandemic in the pre-vaccine era by analyzing de-identified, privacy-preserving location data for a panel of over 5.5 million anonymized, opted-in U.S. devices. We define five indicators of users’ mobility and proximity to investigate how the emerging collective behavior deviates from typical pre-pandemic patterns during the first nine months of the COVID-19 pandemic. We analyze both the dramatic changes due to the government mandated mitigation policies and the more spontaneous societal adaptation into a new (physically distanced) normal in the fall 2020. Using the indicators here defined we show that: a) during the COVID-19 pandemic, collective physical distancing displayed different phases and was heterogeneous across geographies, b) metropolitan areas displayed stronger reductions in mobility and contacts than rural areas; c) stronger reductions in commuting patterns are observed in geographical areas with a higher share of teleworkable jobs; d) commuting volumes during and after the lockdown period negatively correlate with unemployment rates; and e) increases in contact indicators correlate with future values of new deaths at a lag consistent with epidemiological parameters and surveillance reporting delays. In conclusion, this study demonstrates that the framework and indicators here presented can be used to analyze large-scale social distancing phenomena, paving the way for their use in future pandemics to analyze and monitor the effects of pandemic mitigation plans at the national and international levels.

## Author summary

The COVID-19 pandemic resulted in some of the most significant disruptions to collective human behavior. In this study, we quantified the nature and scale of these disruptions during the first nine months of the pandemic by estimating changes in daily routines related to mobility, commuting, and social contacts. We used high-resolution mobility data that describe the physical movements of over 5.5 million individuals in the United States. Our findings indicate that the strength of the behavioral responses varied during different phases of the pandemic, across locations (states and cities), and across social settings (urban versus rural). We also found that reductions in commute flows were correlated with employment characteristics and that our proposed indicators for social interactions could be used as early warnings of potential future negative health outcomes (e.g., new daily deaths), thus opening up the possibility of using these metrics as additional situational awareness tools in future outbreaks.

## Introduction

The near-ubiquity of mobile phone usage—coupled with state-of-the-art techniques for data anonymization and user privacy [1, 2]—has led to unprecedented opportunities to gain insight into the social response to the COVID-19 pandemic [3–11], to improve our understanding of human behavior by quantifying reductions in mobility and changes in consumer behavior [9, 12], and it has contributed to the debate around the effectiveness of the different policies and guidelines introduced to mitigate the spread of the COVID-19 pandemic [13–20].

Here, we present a framework aimed at characterizing collective patterns of physical distancing and we show some of its applications by looking at behavioral changes over time and locations, and by examining the relationship between the observed changes and employment characteristics and health outcomes. The proposed approach consists of several measures of mobility and physical proximity: 1) the daily range of mobility for each user; 2) the fraction of users that commute to work; 3) the fraction of users that travel between metropolitan areas; 4) the number of unique contacts outside of home and work; and 5) the average duration of those contacts. We compute these measures over a sample of anonymized, privacy-preserving aggregated location data for a panel of approximately 5.5M users selected from more than 40 million mobile devices geolocated in the United States between January and September, 2020. Together, these complementary measures provide a macroscopic signature of what happens to a population when millions of individuals reduce their mobility and physical proximity. These measures allow us to provide one possible working definition of collective physical distancing in the United States during the first nine months of the COVID-19 pandemic, pre-vaccine era, and to quantify how it emerged—and, to some extent, persisted—following work-from-home policies, mobility restrictions, shelter-in-place orders, and other policy interventions implemented and promoted during the COVID-19 pandemic [21–23]. We show that the defined measures capture relevant differences of behavior changes in urban versus rural settings and they are statistically associated with unemployment and teleworking rates. Notably, we also find that the measures characterizing reduction in individual contacts are early indicators of COVID-19 deaths. These findings suggest that the proxy measures identified here can, in turn, be used to calibrate epidemic transmission models aimed at defining the burden of the COVID-19 epidemic [3, 24–26]. An interactive version of the results and measures included in this manuscript (as well as access to the anonymized, aggregated dataset) is made publicly available through the following online dashboard: https://covid19.gleamproject.org/mobility.

## Materials and methods

### Mobility data

Mobility data are provided by Cuebiq Inc., a location intelligence and measurement company. Through its Data for Good program (https://www.cuebiq.com/about/data-for-good/), Cuebiq Inc. provides access to aggregated and privacy-enhanced mobility data for academic research and humanitarian initiatives. Cuebiq Inc. collects its data primarily through its proprietary location-based Software Development Kit (SDK) that partners embed in their mobile apps. In other words, Cuebiq Inc. mobility data is collected from smartphone applications where location is at the core of the app’s functionality. This includes app categories such as maps, navigation, weather, and geo-specific retail. All data is collected with the informed consent of fully anonymized users under GDPR and CCPA compliant frameworks. As part of the opt-in process, users consent to share their anonymized data directly with Cuebiq Inc. for research purposes. Users may opt out at any time, request copies of their data, and request that their data be permanently deleted under portability and erasure clauses. In addition to fully anonymizing the device IDs of each user by utilizing an encrypted hash, the mobility data undergoes additional privacy protections by utilizing patented privacy enhancing technologies. First, the inferred coordinates of users’ home and work locations are up-leveled so that the new coordinates will correspond to the centroid of their corresponding Census block group [27], thereby precluding identification of individual users based on home or work addresses data, while preserving sociodemographic inference capabilities based on publicly available census data. Then, the mobility data is also subject to cleansing to remove visits to sensitive points of interest, including military bases, sexual reproductive health centers, places of worship, elementary schools, and other places with heightened levels of privacy sensitivity.

While this mobility dataset has been released to researchers in an effort to assist COVID-19 response and epidemic modeling efforts, the data collection process on itself has not been specifically tailored for public health studies. In this study, we characterize collective physical distancing for a panel of 5,506,590 users that were active between January 7^*th*^ and June 30^*th*^, 2020. Specifically, users are included in the panel if all the following conditions apply. First, each user must be active for at least 21 days in each month from January until June 2020. Second, on average, the spatial coordinates of each user must be recorded at least once per hour (averaged over the number of days in which a user is active). Lastly, the average geolocation accuracy for each device needs to be less than 50 meters for the period of coverage. The considered panel of users is representative of the U.S. population for several socio-demographic characteristics such as age, sex, race, educational attainment, and earnings (see S1 Text).

### Demographic and employment data

County-level demographic data, including the rural-urban designation as well as demographic data used for statistical corrections are from the United States Census and the American Community Survey (https://www.census.gov). County-level unemployment data are from the United States Bureau of Labor Statistics. Data about the percent of teleworkable jobs are from Dingel and Neiman [28] and Dey et al. [29] that provide estimates for the percent of jobs that can transition to telework for Metropolitan Statistical Areas (MSAs) based on their occupation distribution within each region.

### State-level COVID-19 testing data and reopening data

Data about the COVID-19 testing and cases are from the COVID Tracking Project [30], which compiles data directly from state health authorities. Data about the dates that states initially began to reopen was collected from the *New York Times* [23].

### Collective physical distancing indicators

In this section, we introduce five different mobility and proximity indicators that we use to quantify daily changes in collective physical distancing: 1) commute volume, 2) mobility range (radius of gyration), 3) inter-CSA transit, 4) distinct contacts per user, and 5) average contact duration.

#### Estimating short-range traveling using daily commute volume

Daily commute volumes count the total number of home to work trips originating from a given county within 24 hours. Cuebiq Inc. provides a list of obfuscated “personal areas” for each user. Observations geolocated from within these locations are deemed to be coming for either the home or the work location of the individual and are therefore up-leveled to preserve user privacy. That is, these coordinates are aggregated to the centroid of the Census block group level that each observation falls into. In order to quantify the changes in commuting behavior to and from work, we classify personal areas into the home or work location to be able to count commute flows. In particular, we consider the most commonly-visited personal area during nighttime hours (9:00pm—5:00am) as the home location of the user, while the most common non-home personal area visited during daytime hours (9:00am—5:00pm) is classified as the work location of the user. This method is imperfect (i.e., it may obfuscate users who exclusively work night shifts), but it is based on assumptions about the typical worker in the United States. Then, one *commute* is defined as a user visiting their “home” and “work” in a given day. Lastly, in this study we take as reference the definition and location of personal areas as identified in the period immediately prior to the lockdown measures. Therefore, our commute metric reflects changes with respect to the *status quo* existing prior to the COVID-19 pandemic.

#### Estimating mobility range using the radius of gyration

The radius of gyration [31] characterizes the extent of a given user’s trajectory in a single day and its formal definition is:

$$\begin{array}{c} r=\sqrt{\frac{1}{n}\sum_{i=1}^{n}∥{\vec{r}}_{i}-{\vec{r}}_{cm}{∥}^{2}}, \end{array}$$ (1)

where *n* is the user’s number of observations on that day, ${\vec{r}}_{i}$ is the *i*^*th*^ observed position of the user, *i* = 1, 2, …, *n*, and ${\vec{r}}_{cm}=\sum_{i}\;{\vec{r}}_{i}/n$ is the center of mass of the trajectory. This measure gives us a standardized way to tell how far an individual is traveling from their average daily position (center of mass), most likely their home and work locations, in a given day. I.e. a larger radius of gyration corresponds to a trajectory with positions that are further away from the person’s center of mass. The radius of gyration provides us with a way to measure the “characteristic distance” [31] traveled daily by each individual and how their spatial range changed during the different phases of the pandemic. From an epidemiological standpoint, this measure is particularly relevant as reductions in people spatial ranges will reduce the rate at which an epidemic will diffuse between (possibly distant) locations. Lastly, in order to compute typical mobility within a given region, we sum the total daily radius of gyration that by users in that region.

#### Estimating long-range traveling using inter-CSA transit

To study the changes in long-range traveling, we calculated the number of users who visited at least two separate U.S. Census Combined Statistical Areas (CSAs) in a single day. In particular, CSAs are defined so that each CSA groups together neighboring areas that share significant employment interchange and that show a substantial degree of economic or social connection between them, often measured by commuting patterns. In other words, we measure the volume of long-range trips between major metropolitan areas which allows us to capture—among other things—also variations in air traffic and long-range train and road trips.

#### Estimating social mixing using daily contacts

For the purpose of contact tracing, the CDC defined a *close contact* as someone who was “within 6 feet of an infected person for at least 15 minutes” [32]. Using this guidance, we operationalize the definition of contact as two devices being within the same rectangular area of approximately 38m × 20m for at least 15 minutes and we define two measures quantifying contact mixing between individuals. Even though defining contacts in this way can be noisy or imprecise due to the spatial resolution considered, we show in the following that the measures introduced in this section positively correlate with key epidemiological indicators, e.g., new deaths.

The method works as follows. Each time the time-stamped spatial coordinates of a user are recorded, we associate the longitude-latitude coordinates to an 8-character *geohash*. A geohash is a short string of letters and digits that allows to encode coordinates into a hierarchical spatial data structure that tessellates the world surface into a grid. In our case, we consider geohashes at an 8-digits resolution which encode rectangular cells of dimensions that are approximately 38m × 19m at the equator [33]. We define two users to be co-located if they are observed in the same geohash for at least 15 consecutive minutes. For each user, we compute the number of *unique* users that a device is in contact with during a single day and the total dwell time of their daily contacts that fit the above criteria. We average these values across users in a given region to arrive at county, state, and nationwide daily *average contact duration* and *average number of distinct contacts*. Note, this method can record only contacts occurring outside of personal areas due to Cuebiq’s privacy protection procedures that obfuscate the exact coordinates for location events occurring within personal areas (e.g. users’ home and work locations).

#### Typical behavior

In this manuscript, we report the metrics as percent of *typical* activity. We select the period between January 16 and February 28, excluding holidays, as our baseline, therefore defining what constitutes, in the context of this work, *typical behavior*. For each measure, we divide the indicator daily value by its average value during the baseline period for the same day of the week (i.e., Mondays are compared to the average Monday during the baseline period). Therefore, values of 100% denote typical behavior. For example, the timeline of the percent of typical behavior for the commuting indicator at time *t* will be computed as $\frac{C_{t}/N_{t}}{\tilde{C}/\tilde{N}}$ where *C*_*t*_ denotes the number of commutes observed at time *t*, *N*_*t*_ denotes the number of active users at time *t*, $\tilde{C}$ denotes the number of commutes in the baseline reference period, and $\tilde{N}$ denotes the number of active users in the baseline reference period.

### Ethics statement

The details of the IRB/oversight body that provided approval for the research described are: Northeastern University, the Office of Human Subject Research Protection (HSRP), IRB exemption number 20–03-23.

## Results

### Quantifying spatial mobility and social mixing

**Fig 1:**
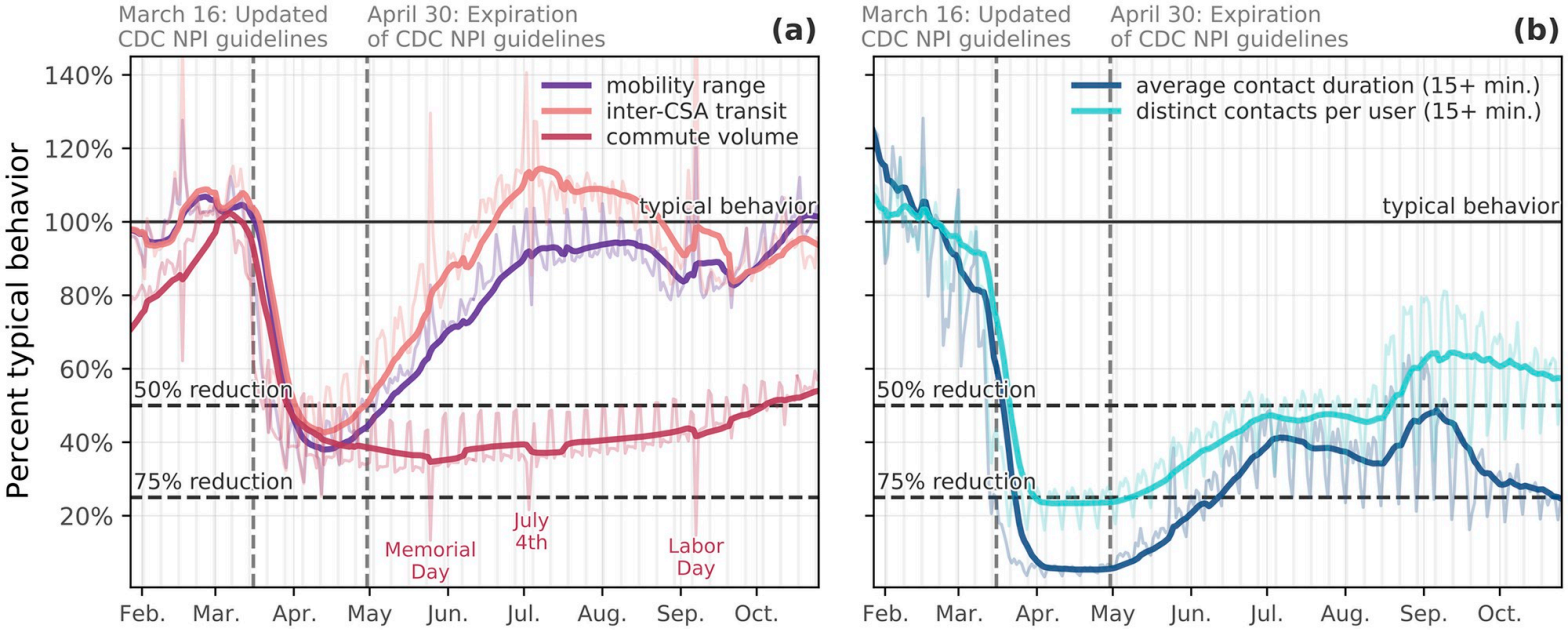
Changes in mobility and social mixing over time. Graphs show deviations from typical behavior for the same weekday in the United States. **(a)** Mobility: Individual mobility (radius of gyration), commute volume, and inter-CSA transit. **(b)** Contacts: Number of distinct contacts and average contact duration events outside of work and home. By the national declaration of emergency (March 13), reductions in spatial mobility measures had begun, reaching approximately 50% of typical values by April 1; while contact measures show a reduction greater than 75% by the same date. A 7-day rolling average is shown alongside each measure. Two grey vertical dashed lines denote the introduction and expiration, respectively, of the CDC non-pharmaceutical interventions guidelines.

On March 16, 2020, the United States government issued guidelines promoting nonpharmaceutical interventions (NPIs) to reduce the spread of the COVID-19 [21]. Such interventions included school closures, state of emergency declarations requiring non-essential businesses to close, and shelter-in-place orders to minimize person-to-person contacts. By April 7, 95% of people in the United States were being urged by their states’ governors to stay home due to the pandemic [22] and by early May 2020, daily commuting volume shows that across the United States there has been a reduction of approximately 65% of the typical daily values (Fig 1a, commute volume). Notably, in our data, the aggregate trend in commute volume has remained relatively stable since early May, at about a 60–70% reduction, though it is beginning to trend upwards again as of early September. We suspect this trend is both a reflection of the reality of the “new normal” of work-from-home policies, along with increases in unemployment due to COVID-19 in the fall of 2020. Commuting behavior informs us about the fraction of individuals that still go to their workplaces and this information is also relevant for the modeling of disease transmission in workplace settings [34–36]. At the same time, we also observe a sharp decline in long-range traveling, as measured by the number of users traveling between CSAs (Fig 1b) as compared to the baseline in every CSA included in this analysis. At its peak, the amount of inter-CSA transits among the users in our panel had decreased by almost 50%, on average.

Lastly, we capture the change in the range of individual daily traveled distance during the COVID-19 pandemic and we show that by early May, the average radius of gyration of users in our panel decreased by between 45–55% relative to a typical weekday, as shown in Fig 1a (mobility range). Similar results have been reported previously for New York City [5]. The range of distance traveled increases steadily throughout May and June, and by early July returns to about 95% of the typical behavior. This increase follows the rescinding of stay-at-home orders and the steady reopening of businesses across the country, meaning increases in mobility for both employees and consumers. However, it is also likely related to increased confidence among the general public that activities requiring traveling, such as trips to the beach and hikes, could be done while practicing social distancing, making them safe to engage in. Indeed, this return to near-typical mobility range is not accompanied by a return to near-typical person-to-person contact events, giving support to the evidence that public’s confidence in the safety of low-contact activities increased in time.

As a first measure of social mixing we considered the number of *distinct contacts* that a user has in a given day, outside of work or home. These contacts quantify the opportunity for disease transmission to/from distinct individuals, being at the same coffee place, interacting at a grocery store, and so on. On average, there was a dramatic decline in the number of distinct contacts that users had in a day with the onset of this decline around March 11 (see Fig 1). Users in our panel had approximately 75% fewer distinct contacts per day by mid-April. Unique contacts increased steadily starting in May and through June, leveling off for the remainder of the summer at approximately 40–50% reduction. This trend reflects a general loosening of physical distancing, consistent with reopening of businesses as well as increased comfort with outdoor gathering. Importantly, however, we do not see a full return to typical behavior, suggesting that even faced with newly reopened amenities (shops, restaurants, etc.) people in the United States remained reluctant to return to pre-pandemic levels of social activity.

Characterizing effective contacts for disease transmission must take into account that the probability of transmission increases also with the duration of the contact [37]. For this reason we measured each user’s average total *duration* of contacts with other users, based on how long their devices were located near each other. The total duration of contacts per day followed a similar pattern to the number of unique contacts. By mid-April, the duration of contacts was reduced by about 75% compared to typical behavior before social distancing measures took effect. Through May and June there was a steady increase up to about a 45% reduction from typical. The fact that total duration of contacts was reduced further than distinct contacts per day indicates that the increase in distinct users met is not always accompanied by an increase in time spent together. Again, some of this could be due to increased comfort with outdoor, socially distanced behavior, such as passing others on a walk through the park. Similar to the mobility range trends discussed earlier, throughout May and June there was a steady increase in contact events. However, the trend does not approach typical behavior by the end of July, instead hovering between 50–65% of typical.

### The phases of collective physical distancing

**Fig 2:**
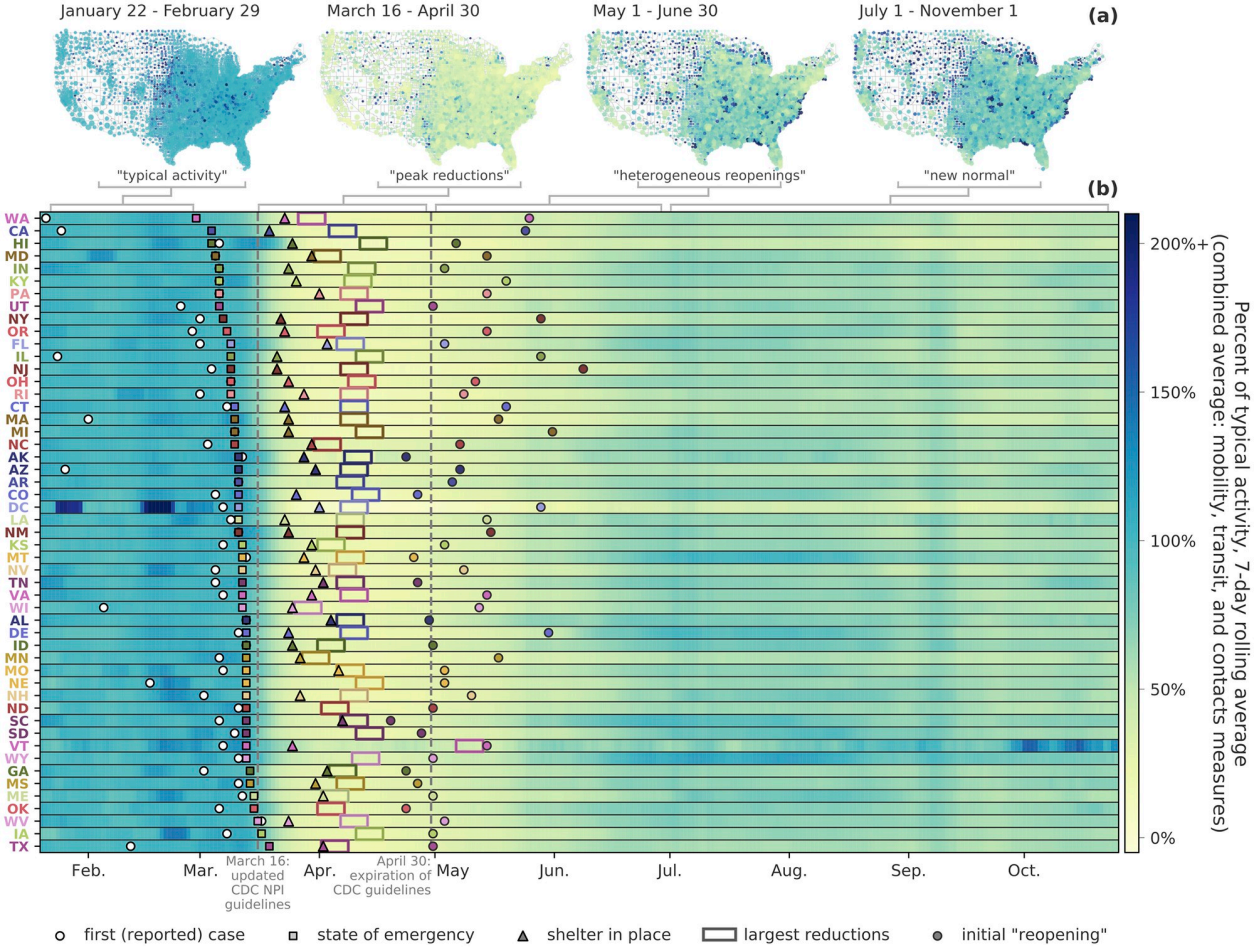
The phases of collective physical distancing in the United States. (Top) County-level maps of collective physical distancing, with each county colored by an average of its typical daily commute volume, individual mobility range, inter-CSA transit, unique contacts outside of home and work, and total duration of contacts for the time frame listed. (Bottom) Heatmap of reductions in contacts and mobility, emphasizing key dates in every state and the key phases of the pandemic in the U.S. (reopening data from [23]).

The COVID-19 pandemic has brought some of the most substantial disruptions to collective human behavior in living memory. The timeline of these behavioral disruptions is clearly visible in Fig 2, where we report an aggregate index for each state computed as the average percentage reduction with respect to the typical activity of the five previously introduced mobility and proximity indicators. Using this aggregate index, we can characterize four distinct phases of collective physical distancing behavior in the United States:

- *Typical activity*. From late January to late February, we observe a baseline period that define the typical activity.
- *Peak reductions*. During this period we observe the dramatic reduction of all indicators following the federal and state mandates and mitigations.
- *Heterogeneous reopenings*. The time window from early May to late June was marked by states’ reopening of businesses and schools according to different schedules and strictness in residual NPI’s.
- *New normal*. From July onward when mobility range and inter-CSA transit increased to values comparable to pre-pandemic levels, while commuting flows (i.e., people going to work) and contact measures generally remained at values lower than typical pre-pandemic levels, characterizing a new stage for people living through a pandemic.

During these four time frames, we see a combination of nationwide reductions in commuting volume to/from work. People’s daily social routines changed dramatically as well, with daily mobility being reduced by up to 60% in April, along with approximately 80% fewer contacts with others per day at the peak of physical distancing. It remains a challenge to identify any single cause of these changes in behavior. However, when looked at together, they offer a way to characterize the evolution of our collective behavior, giving us a baseline for understanding how societies react to such a massive disruption. In the Supporting Information, we provide measure-specific curves for each state (S12 Fig) and for several major metropolitan areas (S13 to S29 Figs).

#### Commuting, working from home, and unemployment

Commuting volume decreased dramatically in early March, and, in most states, did not increase in the same way that the other mobility/contact measures have. This is likely due to several reasons, from the historic waves of unemployment in the spring and early summer, to a dramatic increase in teleworking. Indeed, throughout the COVID-19 pandemic in the U.S., we see a strong negative correlation between the percentage of teleworkable jobs and commute volume in metropolitan statistical areas (MSAs). For public health officials planning for future pandemics, this relationship between telework and commute volume is especially insightful; many of the NPIs that have been introduced throughout the pandemic were designed to limit the amount of workplace infections, and as such, reductions in commute volume that are due to increased telework (as opposed to increased unemployment) could illustrate a preferable balance of economic and public health priorities.

During the COVID-19 pandemic, many employers eliminated in-person interactions, although many jobs in the United States cannot easily transition to remote work [28, 29, 38]. Furthermore, it is difficult to quantify the ubiquity of remote work across the United States and various surveys have been conducted trying to estimate this number [39]. In addition, typical commuting patterns were also impacted by an unprecedented surge in unemployment [40]. Therefore, we explore the relationships between commuting, unemployment, and teleworking by combining our commute metric with two additional data sources: the Bureau of Labor Statistics’s Local Area Unemployment Statistics (LAUS) dataset, which provides monthly estimates of county-level unemployment rates; and Dingel and Neiman’s and Dey et al.’s estimates of the proportion of jobs in an area that can be feasibly worked from home [28, 29]. The teleworkability estimates are at the Metropolitan Statistical Area (MSA) level, and the LAUS data at the county level. For this reason, in our analysis, we aggregate our measurements to the MSA level, excluding rural counties. Note that while local unemployment and commute volume vary month-to-month, the estimated proportion of teleworkable jobs is largely a static quantity.

**Fig 3:**
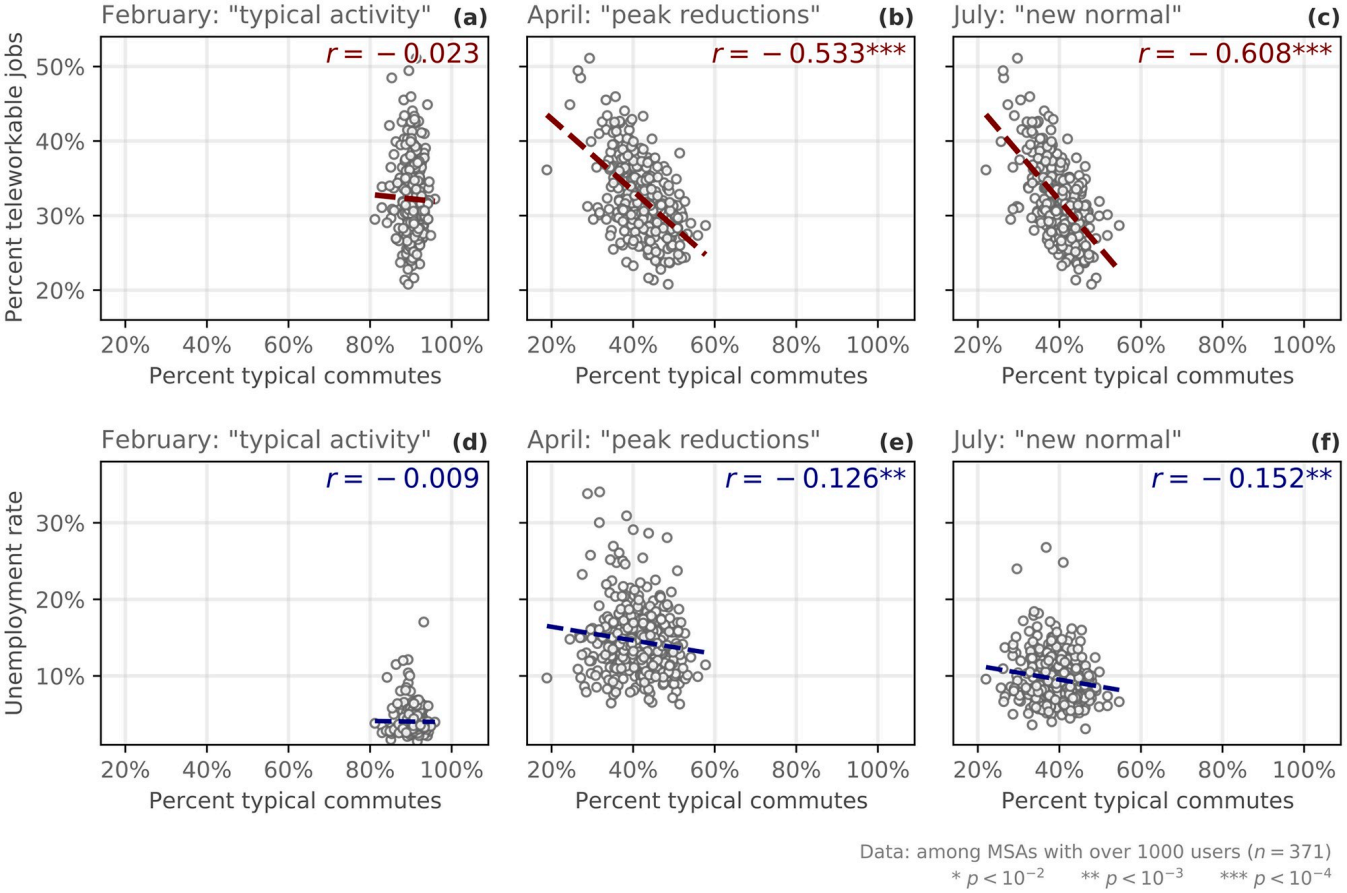
Unemployment, teleworking and commuting patterns. Grouping county-level employment data to the Metropolitan Statistical Area (MSA), we correlate commute volume with the percent of jobs that can readily transition to teleworking according to Dingel and Neiman [28] (top row) and unemployment rate (bottom row) over time. **(a & d)**: February, during the baseline period; **(b & e)**: April, during the peak lockdown; **(c & f)**: July, after unemployment declined but commuting remained low—during the “new normal” phase).

In Fig 3, we present the relationship between commute volume, unemployment, and teleworkability in February, April, and July, with each month corresponding to a distinct phase of the pandemic. In February—the baseline period for which we use to define “typical” activity—there is no correlation between the percent of typical commutes in MSAs and the percent of teleworkable jobs, nor is there a correlation between commuting and unemployment at that point (Fig 3a and 3d). This is expected, as the United States had not yet experienced large disruptions resulting from the COVID-19 crisis. Then, in April, following the updated guidelines about physical distancing and the massive surges in unemployment, commuting volume dropped substantially across the United States (on average, about 40% of baseline levels, see Figs 1a and 3b). During this lockdown period, the percentage of jobs that can transition to telework show a -0.533 correlation with commute volumes (Fig 3b); we also observe a significant negative correlation between commute volume and unemployment rate during this period (Fig 3e).

#### Collective physical distancing in rural and urban areas

**Fig 4:**
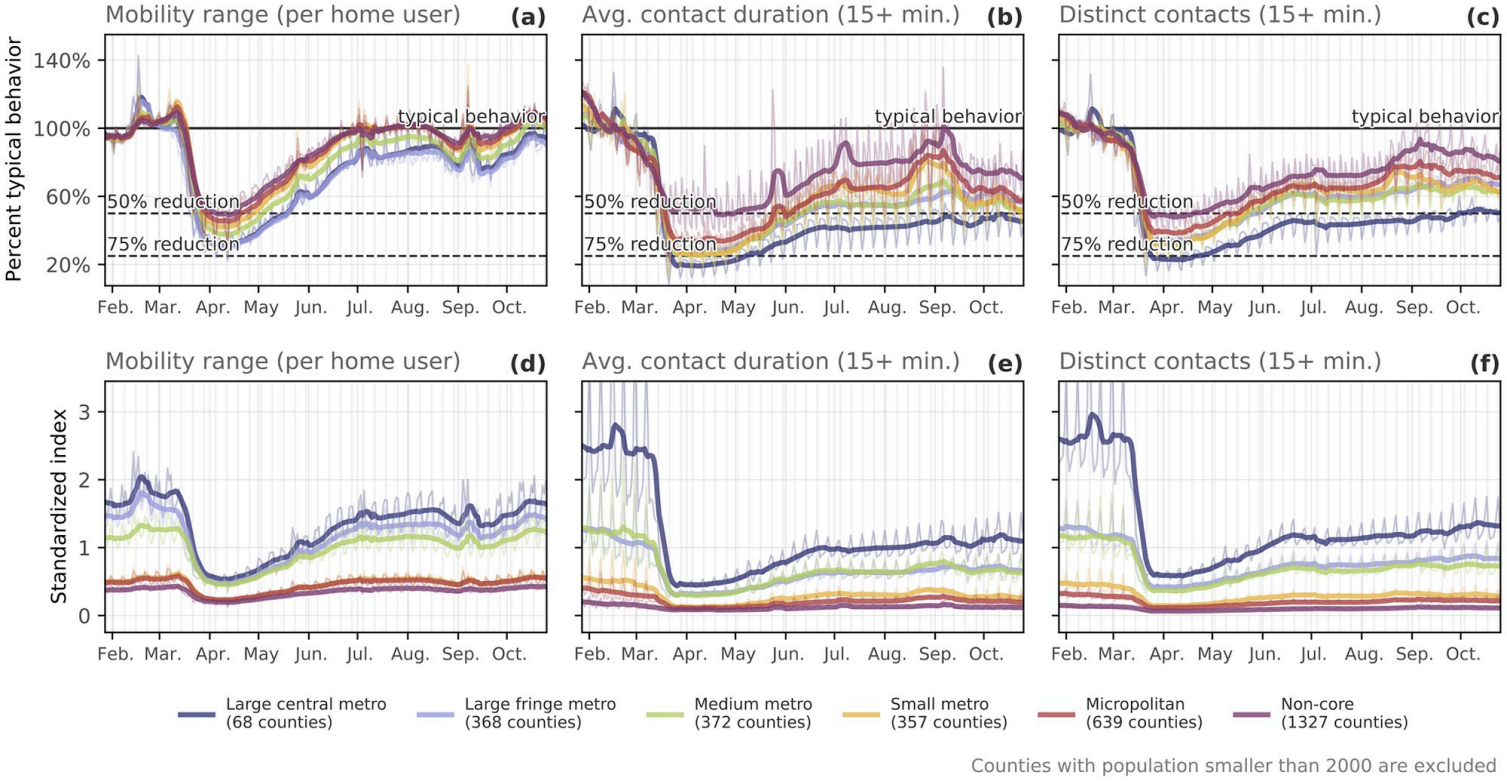
Differences in county-level behavior based on rural-urban codes. Each county in the United States is assigned a rural-urban code, ranging from 1 (large central metro) to 6 (highly rural, “non-core” counties). We average the percent of typical behavior per user (top row) and a standardized index (bottom row) across counties grouped by these six rural-urban code designations. The standardized index obscures raw values but preserves relative differences between groups; we do so by normalizing by the median value across all counties. **(a & d)**: mobility range; **(b & e)**: contact duration; **(c & f)**: distinct contacts. Seven-day rolling averages are plotted in bold above raw values plotted as thin curves.

We also observe different levels of collective physical distancing in different parts of the country, which reflects the heterogeneity in policy response, disease incidence, geography, and population structure across the US (see e.g. [41]). By grouping our collective physical distancing measures with the National Center for Health Statistics (NCHS) urban-rural county classification scheme [42], we can compare the responses of people living in urban versus rural settings. We observe that large central metro (code 1) areas showed the largest reductions in our collective physical distancing measures for contacts and mobility range (Fig 4a–4c); the more rural the county, the less reduction from typical behavior. However, users living in more urban counties also had lower baselines for these measures (Fig 4d–4f), which we show using a standardized index as opposed to the “percent of typical” values shown in Fig 4a–4c. As a point of comparison, by the beginning of April, the median reduction of the number of distinct contacts for users in large/medium metro counties approached a level similar to the baseline of a typical user in rural, micropolitan counties (Fig 4f).

More-urban areas also began physical distancing behaviors earlier than more-rural areas; for example, large central and large fringe metro areas dipped to 80% of typical for their contact measures about five days earlier than small metropolitan areas, micropolitan areas, and non-core areas. During the first week of May, micropolitan and non-core counties showed mobility range that was around 75% of typical, while large central and fringe metro areas remained at around 50% of typical (Fig 4a). A similar rural-urban gap is seen in the percent of typical behavior for both measures of contacts (Fig 4b and 4c).

#### Collective physical distancing and the toll of COVID-19

Lastly, we validate the use of the proximity/contact measures introduced in this manuscript as coarse-grained approximations for true person-to-person contacts. As such, we would expect to find a positive correlation between these measures and key epidemiological indicators, such as new deaths. More specifically, we would expect that a *lagged* correlation would best capture this relationship since it accounts for: a) the time from exposure to symptom onset (about 6 days); b) the median number of days from symptom onset to death (between 13 to 17 days depending on the age group considered); and c) the median number of days from death to reporting date (varying from 19 to 21 days depending on the age group) [43]. Because of these factors, the median delay we would expect in the correlation between our proxies for contacts and new deaths is in the range 38–44 days. Nationwide, we see that this is indeed the case.

**Fig 5:**
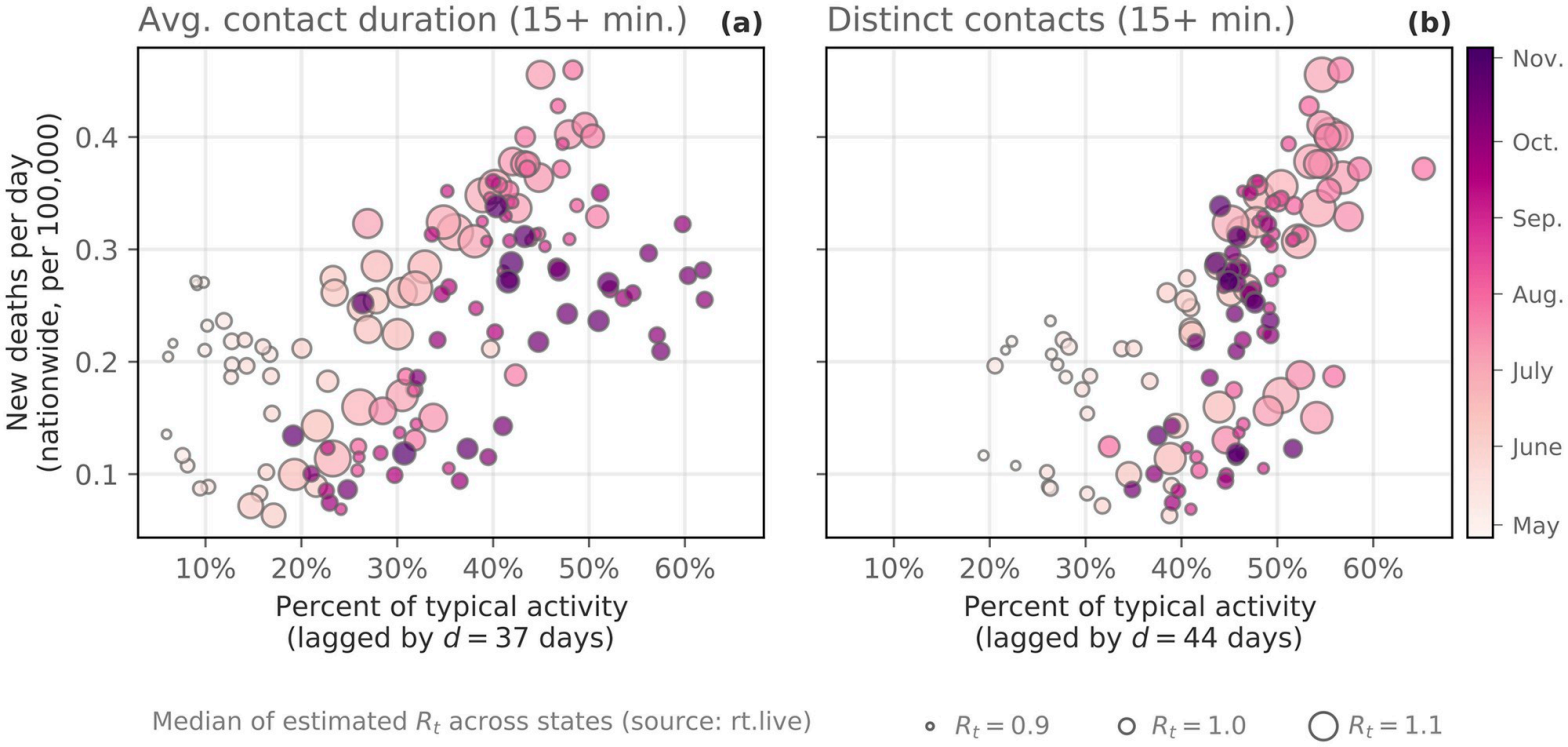
Collective physical distancing and new deaths. Here we correlate daily contact measures nationwide with new reported deaths [30] between April 30 and November 5, 2020. The horizontal axes correspond to the percent of typical contact patterns, while the vertical axis corresponds to the (lagged) number of new deaths per 100,000. **(a)** Average contact duration **(b)** Distinct contacts. A lag of *d* days was selected for each state so as to maximize the correlation between new deaths and contact measures. Maximum correlation is observed at *d* ∈ [37, 44] (*d* = 44 is visualized) days that is consistent with CDC estimates [43] that account for disease dynamics and reporting delays. In each subplot, darker colors indicate later dates and marker size corresponds to an estimate of the median effective reproductive number (*R*_*t*_) across all 50 states and District of Columbia (source: rt.live). These contact measures are also positively correlated with new reported cases (but at a shorter lag, see S11 Fig).

In Fig 5, we plot the nationwide percents of typical average contact duration (a) and distinct contacts (b) against the daily number of new deaths per 100,000 people nationwide. The contact measures are correlated at a delay *d* ∈ [37, 44] days for each measure, which was selected by maximizing the correlation between contact patterns and new deaths. We do the same comparison for the number of new infections in the S4 Text (S11 Fig), highlighting the robustness of this correlation nationwide.

The color of the markers in Fig 5 corresponds to time; darker colors indicate later dates. Here, we see an important relationship between our contact measures and the course of the pandemic. Namely, as contact patterns increased in the early summer (lighter colored markers), new infections and new deaths followed; this, in turn, was followed by *decreases* in contact events, followed again by decreases in new infections by late August (darker colored markers). This is approximately the same time as when the curves in Fig 1b (the contact measures) started to level off, while mobility and inter-city transit continued to rise (Fig 1a). What this disconnect between mobility and contact patterns suggests is that our collective social behavior can reduce the rate of new infections and, as a result, new deaths. This finding is possibly trivial to epidemiologists and public health officials, but it is nonetheless important for our understanding of how our collective behavior impacts the trajectory of a pandemic, to validate our contacts measures as proxies for true person-to-person contacts, and it is also consistent with other findings throughout the literature on COVID-19 [11, 44–46]. The ability to measure these patterns in almost real time shows the potential benefits of using mobile device data in forecasting (or “nowcasting”) the trajectory of a virus, and moving forward, they present a baseline for our collective behavioral response to future pandemics.

## Discussion

The massive efforts to comply with the CDC’s physical distancing guidelines came at a substantial cost to the economic and social well-being of people in the United States. By quantifying these nationwide behavioral changes, we get a glimpse into the relationship between large scale collective behavior and the course of the pandemic. Learning from these patterns is necessary to prepare for future pandemics; most notably because despite large-scale collective physical distancing, during the time window from February to December 2020, the United States reported over 13 million cases of COVID-19. Furthermore, considering the period up to December 31st, 2020, more than 385,000 deaths were attributed to COVID-19 on death certificates [47]. This suggests that in the pre-vaccine era, the timing, magnitude, and synchrony [48, 49] of collective physical distancing in the United States was ultimately insufficient to completely mitigate the nationwide outbreak. However, studies have shown that combining social distancing with other interventions such as extensive testing, quarantines, and contact-tracing can help to keep the epidemic incidence to lower levels thus helping the local healthcare systems, while allowing for a certain degree of relaxation of social distancing measures [34]. Indeed, that was the approach that some countries, such as South Korea, Taiwan, and China had followed [50–52]. Ultimately, for such a combined approach to work, it is essential to be able to quantify collective social distancing utilizing near-time indicators, like the ones discussed in this work.

During the “new normal” period from July to December 2020, there were millions of new cases and hundreds of thousands of new deaths in the United States; during this same time period, we see mobility patterns return to 100% of baseline levels while contacts remained at around 65% of typical activity. This suggests two key things. First, a national average of approximately 65% of typical contacts was not sufficient for avoiding the large number of cases seen during that period. Further modeling efforts are needed to estimate the potential effects that larger decreases in contacts would have had (e.g. 60%, or 50%, etc. instead of 65%). Second, this suggests that over the course of the pandemic, people may have learned to adapt their behavior in a way that allows them to travel while still limiting opportunities for contact with others. For example, visiting a park or hiking are activities that are likely associated with higher mobility but not necessarily more contacts. Indeed, in many cities across the United States, we see a relative rise in visits to parks [9] during this time period. Learning from this might inform goals or benchmarks for policy responses to this or future pandemics.

In this work, we quantified the unprecedented behavioral response to COVID-19 in the first 9 months of the COVID-19 pandemic in the United States—collective physical distancing at a nationwide scale—using five different measures of mobility and contact patterns. By studying the daily mobility patterns of millions of anonymous mobile phone users, we show how people altered their typical behavior, limiting daily interactions with others to comply with policy interventions and in an effort to reduce their chances of becoming infected with the virus. Understanding precisely and quantifying how individuals’ behavior changed over the course of the pandemic is critical, and in this work we present several measures that transform large-scale mobile device data into near real-time epidemiological insights. Of particular importance, the contact proximity measures introduced here correlate with the onset of new deaths nationwide; this correlation is maximized at a delay of 37–44 days, in line with the range reported by the CDC [43].

Recent work has shown that a more nuanced understanding of typical human mixing patterns can have dramatic effects on the spread of a disease and our models of the spread of a disease; it is particularly useful to understand age-based, setting-specific contact patterns within a population [36, 53]. The current study is limited by the absence of this data, and in many ways traditional surveying methods may offer more robust estimates (see [53]). However, the measures of collective physical distancing behavior that we introduce can be potentially generalized by using differences in Census tracts age distributions to estimate (on aggregate) age-specific mobility and contact reductions. Lastly, we quantify contacts based on geographic proximity and we do not attempt to link locations to information about the *setting* where these contacts take place in (i.e., at a restaurant, workplace, park, etc); this information is particularly relevant because the odds of disease transmission are much higher with contacts in closed spaces compared to open-air environments [35, 54]. This can be addressed by measuring contact events within a pre-identified list of points-of-interest.

Cuebiq Inc.’s mobility data used in this work have both strengths and weaknesses. As shown in this study, it is clear that this data can play a significant role in enriching our understanding of the effects of policy interventions and therefore enhance our ability to realistically model and predict disease spreading during an ongoing outbreak by providing us with a real-time situational awareness tool that can allow us to monitor changes in physical behaviors and mobility. However, these data also have some limitations: reliability may be impacted by the user opt-out mechanism if a significant number of users decide to opt-out of the data sharing agreement; we do not have information regarding the specific list of apps the Cuebiq users’ are utilizing and therefore we cannot directly control for potential self-selection biases in the user population; GPS data can sometimes be imprecise, particularly in densely populated urban areas or locations with poor GPS signal, which could affect the quality of location-based data; and the generalizability of the data might be limited due to its collection from apps where location is central to functionality. In this study, we addressed the data limitations by building a selected panel of users and by assessing its representativeness with respect to key sociodemographic characteristics. Nevertheless, we have shown that it is possible to quantify collective physical distancing, at-scale, during an ongoing pandemic using high-resolution location data. Moving forward and despite the limitations listed above, the use of mobility and proximity indicators like the ones proposed in this work will enable us to devise more precise and effective mitigation strategies, allowing for the possibility of integrating social distancing approaches with other interventions, and ultimately informing our actions and policies in the face of future pandemics.

## Supporting information

S1 Text Panel representativeness. (PDF)

S2 Text Correlating physical distancing measures across datasets. (PDF)

S3 Text Sensitivity analysis: Teleworkable jobs and commuting. (PDF)

S4 Text Correlating contact patterns with new positive tests. (PDF)

S5 Text Citation diversity statement. (PDF)

S1 Fig Panel membership coverage. (PDF)

S2 Fig National Level Socio-Demographics. (PDF)

S3 Fig County-level weights. (PDF)

S4 Fig Schematic of statistical procedure for assigning county-level weights. (PDF)

S5 Fig Changes in mobility and person-to-person contacts over time (unweighted panel). (PDF)

S6 Fig Collective physical distancing and new deaths (unweighted panel). (PDF)

S7 Fig Collective Physical Distancing: Weighted vs unweighted. (PDF)

S8 Fig Correlations across mobility datasets. (PDF)

S9 Fig Comparison across mobility datasets. (PDF)

S10 Fig Teleworking and commuting patterns. (PDF)

S11 Fig Collective physical distancing and new infections. (PDF)

S12 Fig Collective physical distancing across every state. (PDF)

S13 Fig Changes in mobility and person-to-person contacts over time in Atlanta–Athens-Clarke County–Sandy Springs, GA-AL. (PDF)

S14 Fig Changes in mobility and person-to-person contacts over time in Boston-Worcester-Providence, MA-RI-NH-CT. (PDF)

S15 Fig Changes in mobility and person-to-person contacts over time in Chicago-Naperville, IL-IN-WI. (PDF)

S16 Fig Changes in mobility and person-to-person contacts over time in Dallas-Fort Worth, TX-OK. (PDF)

S17 Fig Changes in mobility and person-to-person contacts over time in Denver-Aurora, CO. (PDF)

S18 Fig Changes in mobility and person-to-person contacts over time in Detroit-Warren-Ann Arbor, MI. (PDF)

S19 Fig Changes in mobility and person-to-person contacts over time in Los Angeles-Long Beach, CA. (PDF)

S20 Fig Changes in mobility and person-to-person contacts over time in Miami-Port St. Lucie-Fort Lauderdale, FL. (PDF)

S21 Fig Changes in mobility and person-to-person contacts over time in New Orleans-Metairie-Hammond, LA-MS. (PDF)

S22 Fig Changes in mobility and person-to-person contacts over time in New York-Newark, NY-NJ-CT-PA. (PDF)

S23 Fig Changes in mobility and person-to-person contacts over time in Orlando-Lakeland-Deltona, FL. (PDF)

S24 Fig Changes in mobility and person-to-person contacts over time in Philadelphia-Reading-Camden, PA-NJ-DE-MD. (PDF)

S25 Fig Changes in mobility and person-to-person contacts over time in Phoenix-Mesa, AZ. (PDF)

S26 Fig Changes in mobility and person-to-person contacts over time in San Jose-San Francisco-Oakland, CA. (PDF)

S27 Fig Changes in mobility and person-to-person contacts over time in Seattle-Tacoma, WA. (PDF)

S28 Fig Changes in mobility and person-to-person contacts over time in St. Louis-St. Charles-Farmington, MO-IL. (PDF)

S29 Fig Changes in mobility and person-to-person contacts over time in Washington-Baltimore-Arlington, DC-MD-VA-WV-PA. (PDF)
