# Supplementary material for: Characterizing collective physical distancing in the U.S. during the first nine months of the COVID-19 pandemic
Source: PLOS Digit Health. 2024 Feb 6;3(2):e0000430. doi: 10.1371/journal.pdig.0000430 (PMC10846712; doi:10.1371/journal.pdig.0000430)
Supplement: S1 Text — (PDF) [file pdig.0000430.s001.pdf]

Panel representativeness

The use of a convenience sample of mobile users to study behavioral variations in time requires caution [1], and we took measures to reduce two major sources of potential bias: user attrition and user selection bias. In particular, first, we measure five collective physical distancing metrics using a stable longitudinal panel of Cuebiq Inc. users who were consistently active between January and June, 2020. Second, we have checked the socio-demographic representativeness of the panel and conducted two separate analyses with and without county-specific sampling weights that control for aggregated socio-demographic characteristics such as age, sex, race, educational attainment, and earnings. Our approach allowed us to create a statistically representative and stable sample of users to adequately measure collective physical distancing in the United States at national, state, and metropolitan levels of aggregation. In addition, as a robustness check, we provide the correlations between our measures of collective physical distancing with several other datasets that have been made publicly available (see Supporting Information). In this comparison, we observe the expected correlations between similarly defined measures. For example, the reduced mobility trends captured in our measures are strongly correlated with Google's stay-at-home measure (Pearson $r = -0.78$ for mobility range, $r = -0.73$ for commute volume, $r = -0.72$ for contact duration, $r = -0.69$ for distinct contacts, and $r = -0.60$ for inter-city transit) in addition to other comparable transit measures (see S8 Fig and S9 Fig for in-depth comparisons and correlations between our measures and those from Apple, Google, Waze, PlaceIQ, and the U.S. Department of Transportation). Critically, the measures proposed in this work provide additional insights that

do not seem to be fully captured by other publicly available datasets, especially when looking at publicly available proxies for human interactions. Let us define the coverage c_i for a generic county i as $c_i = n_i/N_i$, where n_i denotes the number of users in our panel that have their home located in county i, while N_i denotes the number of people living in county i according to the official U.S. Census statistics. Then, in S1 Fig, we show that while the median of the distribution of the c_i's coverages is only about 1.76%, the overall correlation between the number of panel users per county and the actual county population is 96.7%. In other words, there is a strong correlation between the number of users observed in our panel for each county and the actual county-level populations, therefore suggesting that our panel does not have strong sampling biases as far as county population size is concerned.

To further test whether our panel provides a good representation of the U.S. population, we compute several socio-demographics characteristics aggregated at the national level from county-level data with each county's contribution proportional to its Cuebiq panel size and we compare such values with the ones obtained where, instead, we use the groundtruth population size for each county. In S2 Fig, we show that our panel displays a high degree of agreement with the values of the statistics as recorded by the 2014-2018 5-year American Community Survey (ACS). In particular, we refer to this version of the panel as the *unweighted panel*, as no sampling weights are applied to individual users. Lastly, as an additional robustness check, we introduce a second version of our panel, the *weighted panel*, in which sampling weights (also known in the literature as survey weights) are assigned to each county to adjust the contribution of each user living in a given county to the estimates of the population socio-demographics characteristics. In particular, using an approach grounded in well-established statistical techniques [2, 3], we estimate sampling weights so that every user's influence on

the estimation of population parameters is proportional to its representation in the population and so that potential user selection bias in the mobility data is reduced. More specifically, we focus on the selection bias that might exist at a sub-national level in terms of age, sex, race, educational attainment, and earnings ("demographics" or "socio-demographic characteristics"). In practice, as an additional robustness check, with the *weighted panel* we adjust all measures presented in this manuscript using county-specific sampling weights, which allow us to create more statistically representative and, thus, more generalizable inferences from our data at the state and national levels of aggregation. Specifically, when aggregating the indexes at the state or national resolutions using the weighted panel, we weigh the mobility and proximity metrics computed for each county, e.g., commutes, not only by the number of users present in our panel for a given location but also by a weight that corrects for the potential selection bias resulting from under- or over-sampling of users with certain socio-demographic characteristics. The weights are estimated using the method outlined in [3], which allows us to estimate the parameters of a target population using data from a potentially biased sample, provided that the determinants of the selection bias are available for both the target population and the sample. In the context of this study, we are estimating mobility and mixing patterns of the US population (i.e., the target population) using our panel data. We are assuming that users' socio-demographics characteristics influence their probability of inclusion in the Cuebiq sample. Therefore, we use information about the distribution of these demographics in both the U.S. population and our panel of users to compute bias-reducing weights. The schematic of the adopted statistical procedure is provided in S4 Fig and described in details in the following.

First, we associate each user to a Census tract by using the location of their home personal area. This allows us to assign to each user a probability distri-

bution of their socio-demographic characteristics by looking at their empirical distributions as reported for each Census tract in the 2014-2018 5-year American Community Survey (ACS) data [4]. Second, we create a synthetic population of users in each census tract, which is a sub-unit of a county, using tract-level data from the ACS. The synthetic population's size in each tract is determined by the number of panel users that are assigned to each tract. Then, to each user we randomly assign age, sex, race, educational attainment, and earnings by sampling their values from the census data. After generating one synthetic population, we compute the mean age, the proportion of males, mean earnings, the proportion of having a college degree or higher, and a proportion of white users for each synthetic county. This process is then repeated 10,000 times, therefore generating 10,000 synthetic datasets. Third, we use a generalized linear model (GLM) with a binomial link function (logit) to estimate the probability that a given county is a synthetic county (as generated from the sample of panel users) or a "census" county where for the given county we directly use the census mean values for the different indicators. In other words, we treat this problem as a classification problem where our regression uses the computed county-specific socio-demographic summary statistics to predict whether a county has been simulated using the Cuebiq sample or not. The intuition behind this procedure is that if our sample is unbiased in terms of the demographics, then the demographic information should not allow us to predict whether a county is a synthetic county or a "census" county (in which case, all estimated β coefficients should not be statistically significantly different from zero). Conversely, if the sample is biased, demographics will produce meaningful predictions.

Using this family of GLMs in the process of reducing sampling bias is standard in well-established techniques such as inverse probability weighting and propensity score matching [2, 5]. To estimate county-specific weights that reduce

bias at the national level, accounting for state effects, we use the following model specification:

$$
\begin{aligned}
P(\text{synthetic}) =& \beta_1 \times \text{age} + \beta_2 \times \text{college} + \beta_3 \times \text{college}^2 + \\
& \beta_4 \times \text{earnings} + \beta_5 \times \text{white} + \beta_6 \times \text{male} + \{\text{state}_s\}_{s=1}^{50};
\end{aligned} \tag{1}
$$

where *age* denotes the county mean age, *college* denotes the proportion of the total population having a college degree or higher, *white* denotes the proportion of the total population being white, *earnings* denotes the average earnings, *male* denotes the proportion of males in a county, and $state_s$ is a state-specific fixed effect. All variables are z-score standardized.

Lastly, we fit our statistical models to each one of the 10,000 synthetic datasets, compute the probability that a given county is a "synthetic" county ($p_{\text{synthetic}}$), and convert it into a county-specific weight w_c^i using:

$$
w_c^i = \frac{1}{p_{\text{synthetic}}} - 1, \tag{2}
$$

where i denotes the dataset used and c denotes the county [3]. We then obtain our final county-specific weight $\bar{w}_c$ as the average of all the estimated weights: $\bar{w}_c = \sum_{i=1}^{I} w_c^i / I$ where I =10,000 (see S3 Fig).

To evaluate the effects of the weighted resampling procedure, we replicate some of the results present in the main text using the unweighted panel. In particular, in S5 Fig we show the changes in mobility and contacts over time, and in S6 Fig we report the correlation between our measures of collective physical distancing and new deaths. In both cases, results are in line with the ones obtained using the weighted sampling procedure. In S7 Fig, instead, we show the effect of using the unweighted vs the weighted panel in computing the time series of the average collective physical distancing (as defined in Fig 2) for each

state. While there are slight differences in some states, the overall picture and insights from our analysis do not change.

Lastly, while we report the results for Vermont in this study, the limited coverage (less than %0.01) prevents us for considering our mobility and contact estimates to be accurate for the state.

References

1. Buckee CO, Balsari S, Chan J, Crosas M, Dominici F, Gasser U, et al. Aggregated mobility data could help fight COVID-19. Science. 2020;368(6487):145–146. doi:10.1126/science.abb8021.

2. Curtis LH, Hammill BG, Eisenstein EL, Kramer JM, Anstrom KJ. Using inverse probability-weighted estimators in comparative effectiveness analyses with observational databases. Medical Care. 2007; p. S103–S107. doi:10.1097/MLR.0b013e31806518ac.

3. Haneuse S, Schildcrout J, Crane P, Sonnen J, Breitner J, Larson E. Adjustment for selection bias in observational studies with application to the analysis of autopsy data. Neuroepidemiology. 2009;32(3):229–239. doi:10.1159/000197389.

4. United States Census Department. American Community Survey; 2018. `https://www.census.gov/programs-surveys/acs`.

5. Dehejia RH, Wahba S. Propensity score-matching methods for nonexperimental causal studies. Review of Economics and Statistics. 2002;84(1):151–161. doi:10.1162/003465302317331982.
