# Supplementary material for: Characterizing collective physical distancing in the U.S. during the first nine months of the COVID-19 pandemic
Source: PLOS Digit Health. 2024 Feb 6;3(2):e0000430. doi: 10.1371/journal.pdig.0000430 (PMC10846712; doi:10.1371/journal.pdig.0000430)
Supplement: S2 Text — (PDF) [file pdig.0000430.s002.pdf]

Correlating physical distancing measures across datasets

In this work, we use data from Cuebiq Inc., but one feature of the COVID-19 pandemic is that mobile providers and other large technology companies have been providing access to aggregated measures of mobility and contacts. For this reason, we include here a series of correlations between the measures studied here and those from a number of other platforms. As a proof-of-principle validation, the measures we include strike a key balance between correlating with existing publicly-available mobility measures (e.g. Google's "residential" measure negatively correlates with each of our measures—which makes sense, as we do not use location pings from within users' home locations) and still providing unique information. The datasets included in S8 Fig and S9 Fig are from: Google (`https://www.google.com/covid19/mobility/`), Apple (`https://covid19.apple.com/mobility/`), PlaceIQ (`https://www.nber.org/papers/w27560`), Waze (`https://www.waze.com/covid19`), and the U.S. Bureau of Transportation Statistics (`https://www.bts.gov/covid-19`).

Broadly, there is correspondence between the measures introduced here and those used by Apple, Google, PlaceIQ, and the U.S. Dept. of Transportation (S8 Fig). The measures that we expect to be highly correlated are indeed highly correlated: for example, Google's "workplace" measure and our commute volume are Pearson correlated at 0.87. Similarly, our mobility measure is highly correlated with Google's and Apple's "transit" measures, and it is negatively correlated with Google's "residential" measure.

Another point of validation can be seen when comparing the various time series of activity (S9 Fig). For major holidays, where we would expect movement to be disrupted, we see broad alignment between the various measures. For example, in early September (Labor Day), we see an increase in the Dept. of Transportation's "Number of trips 100 miles+" measures; similarly we see the same spike in our inter-city transit measure.

There are endless ways to compare the myriad measures of mobility that have been studied during the COVID-19 pandemic, and despite this, the measures included in this work are balanced between offering a novel, informative lens to understand collective physical distancing while also corresponding neatly to measures that have already been proposed in the literature.
