# Supplementary material for: Characterizing collective physical distancing in the U.S. during the first nine months of the COVID-19 pandemic
Source: PLOS Digit Health. 2024 Feb 6;3(2):e0000430. doi: 10.1371/journal.pdig.0000430 (PMC10846712; doi:10.1371/journal.pdig.0000430)
Supplement: S3 Text — (PDF) [file pdig.0000430.s003.pdf]

Sensitivity analysis: teleworkable jobs and commuting

In S10 Fig, we show that the relationship between commute volumne and teleworkability is maintained when using Dey et al.'s revised estimates on teleworkable jobs [1].

References

1. Dey M., Frazis H., Piccone D.S. Jr, Loewenstein M.A. Teleworking and lost work during the pandemic: new evidence from the CPS Monthly Labor Review, U.S. Bureau of Labor Statistics; 2021.
