# Supplementary material for: Characterizing collective physical distancing in the U.S. during the first nine months of the COVID-19 pandemic
Source: PLOS Digit Health. 2024 Feb 6;3(2):e0000430. doi: 10.1371/journal.pdig.0000430 (PMC10846712; doi:10.1371/journal.pdig.0000430)
Supplement: S4 Text — (PDF) [file pdig.0000430.s004.pdf]

Correlating contact patterns with new positive tests

The contact measures introduced in this manuscript are meant to be used as a lower resolution approximation for true person-to-person contacts in the U.S. population. As we showed, the average contact duration and distinct contact measures both are positively correlated with (lagged) new reported deaths. In S11 Fig, we show that this pattern also holds for new positive tests at the national level and for most states (data from the COVID Tracking Project [1]). Again, we plot the lagged correlation that maximizes the R-squared; in this case, the delay that maximizes the average contact duration is 9 days, while distinct contacts is maximized at $d = 14$ days. While testing data is typically noisier and depends on local testing policies, the presence of these positive correlations between contact patterns and lagged new cases is again suggestive that the measures used in this work can serve as coarse indicators of large-scale human behavior.

References

1. Miller K, Curry K. The COVID Tracking Project; 2020. `https://github.com/COVID19Tracking`.
