# Supplementary material for: Characterizing collective physical distancing in the U.S. during the first nine months of the COVID-19 pandemic
Source: PLOS Digit Health. 2024 Feb 6;3(2):e0000430. doi: 10.1371/journal.pdig.0000430 (PMC10846712; doi:10.1371/journal.pdig.0000430)
Supplement: S5 Text — (PDF) [file pdig.0000430.s005.pdf]

Citation diversity statement

Recent work has quantified bias in citation practices across various scientific fields; namely, women and other minority scientists are often cited at a rate that is not proportional to their contributions to the field [1–8]. In this work, we aim to be proactive about the research we reference in a way that corresponds to the diversity of scholarship in this field. To evaluate gender bias in the references used here, we obtained the gender of the first/last authors of the papers cited here through either 1) the gender pronouns used to refer to them in articles or biographies or 2) if none were available, we used a database of common name-gender combinations across a variety of languages and ethnicities. By this measure (excluding citations to datasets/organizations, citations included in this section, and self-citations to the first/last authors of this manuscript), our references contain 3% woman(first)-woman(last), 28% woman-man, 8% man-woman, 60% man-man, 0% nonbinary, 3% man solo-author, and 0% woman solo-author. This method is limited in that an author's pronouns may not be consistent across time or environment, and no database of common name-gender pairings is complete or fully accurate.

References

1. Zurn P, Bassett DS, Rust NC. The citation diversity statement: A practice of transparency, a way of life. Trends in Cognitive Sciences. 2020;24(9):669–672. doi:10.1016/j.tics.2020.06.009.

2. Dworkin JD, Linn KA, Teich EG, Zurn P, Shinohara RT, Bassett DS. The extent and drivers of gender imbalance in neuroscience reference lists. Nature Neuroscience. 2020;23(8):918–926. doi:10.1038/s41593-020-0658-y.

3. Chakravartty P, Kuo R, Grubbs V, McIlwain C. #CommunicationSoWhite. Journal of Communication. 2018;68(2):254–266. doi:10.1093/joc/jqy003.

4. Maliniak D, Powers R, Walter BF. The gender citation gap in international relations. International Organization. 2013;67(4):889–922.

5. Dion ML, Sumner JL, Mitchell SML. Gendered citation patterns across political science and social science methodology fields. Political Analysis. 2018;26(3):312–327. doi:10.1017/pan.2018.12.

6. Caplar N, Tacchella S, Birrer S. Quantitative evaluation of gender bias in astronomical publications from citation counts. Nature Astronomy. 2017;1. doi:10.1038/s41550-017-0141.

7. Azoulay P, Lynn F. Self-citation, cumulative advantage, and gender inequality in science. Sociological Science. 2020;7. doi:10.15195/v7.a7.

8. Ghiasi G, Mongeon P, Sugimoto CR, Larivière V. Gender homophily in citations. 23rd International Conference on Science and Technology Indicators. 2018; p. 1519–1525.
