# Supplementary material for: Characterizing collective physical distancing in the U.S. during the first nine months of the COVID-19 pandemic
Source: PLOS Digit Health. 2024 Feb 6;3(2):e0000430. doi: 10.1371/journal.pdig.0000430 (PMC10846712; doi:10.1371/journal.pdig.0000430)
Supplement: S1 Fig — (PDF) [file pdig.0000430.s006.pdf]

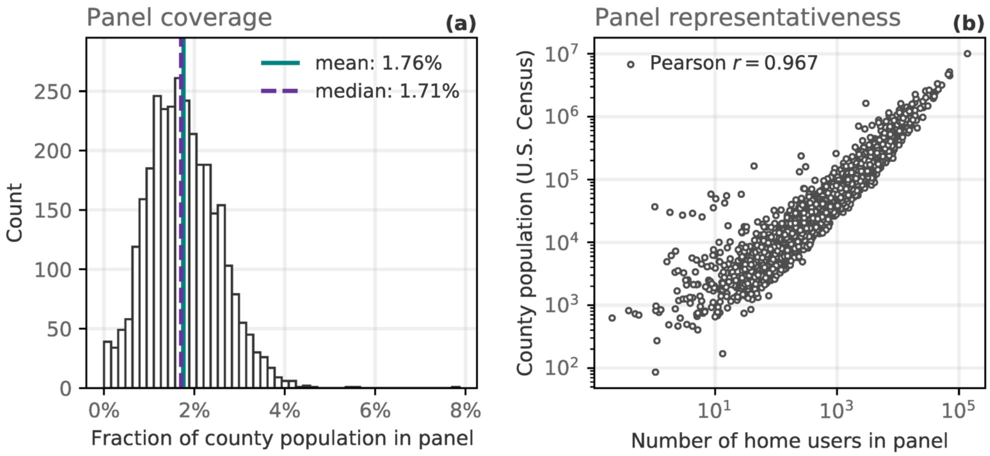

S1 Fig. Panel membership coverage. By inferring counties of users' "home" personal areas from the data, we can see the extent to which we are over/under-representing users on a per county basis. **(a)** Histogram of the fraction of population included in our panel of users for each county. **(b)** Scatterplot correlating the number of home users in a county against the total population of the county.
