# Supplementary material for: Characterizing collective physical distancing in the U.S. during the first nine months of the COVID-19 pandemic
Source: PLOS Digit Health. 2024 Feb 6;3(2):e0000430. doi: 10.1371/journal.pdig.0000430 (PMC10846712; doi:10.1371/journal.pdig.0000430)
Supplement: S2 Fig — (PDF) [file pdig.0000430.s007.pdf]

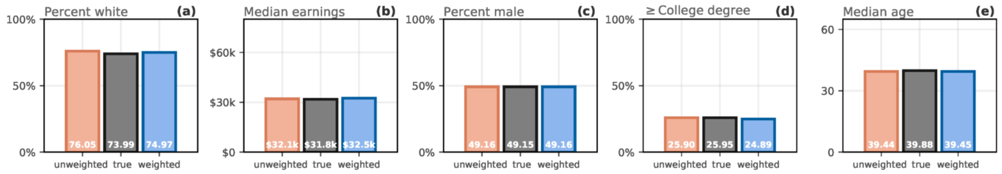

S2 Fig. National Level Socio-Demographics. True values for five national level socio-demographic characteristics – the proportion of males, median earnings, the proportion of having a college degree or higher, and a proportion of white users – as recorded by the 2014-2018 5-year American Community Survey (ACS) data and their aggregated reconstructed values when considering the unweighted and weighted versions of the Panel of users.
