# Supplementary figures and images for: Characterizing collective physical distancing in the U.S. during the first nine months of the COVID-19 pandemic
Source: PLOS Digit Health. 2024 Feb 6;3(2):e0000430. doi: 10.1371/journal.pdig.0000430 (PMC10846712; doi:10.1371/journal.pdig.0000430)

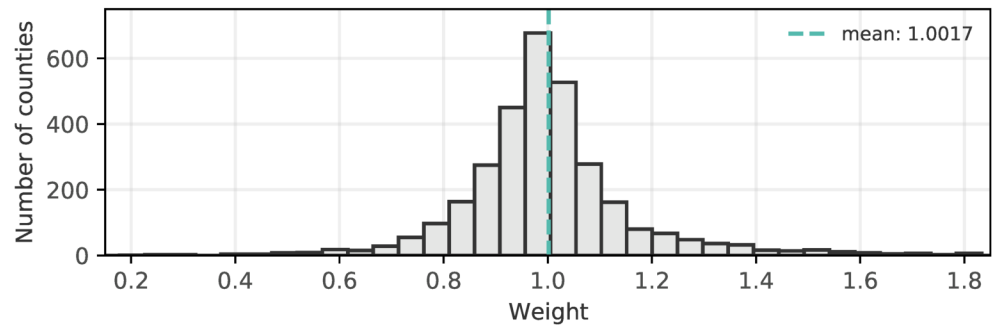

**S3 Fig. County-level weights.** Distribution of county-specific sampling weights  $\bar{w}_c$ 's.

Supplement: S3 Fig — (PDF) [file pdig.0000430.s008.pdf]
