# Supplementary material for: Characterizing collective physical distancing in the U.S. during the first nine months of the COVID-19 pandemic
Source: PLOS Digit Health. 2024 Feb 6;3(2):e0000430. doi: 10.1371/journal.pdig.0000430 (PMC10846712; doi:10.1371/journal.pdig.0000430)
Supplement: S4 Fig — (PDF) [file pdig.0000430.s009.pdf]

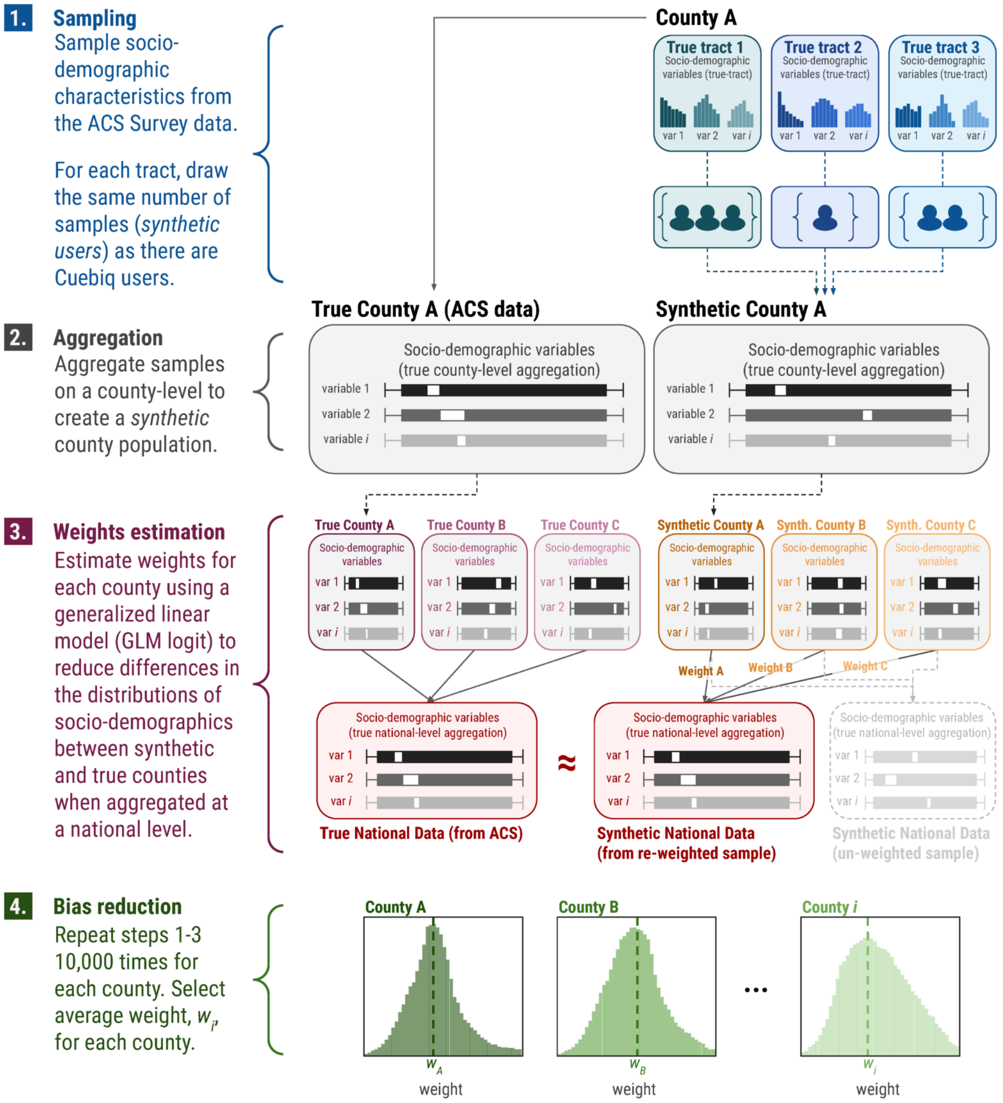

S4 Fig. Schematic of statistical procedure for assigning county-level weights. Through repeatedly simulating synthetic populations at the *census tract* level (based on the number of Cuebiq users with "home" personal areas in each census tract), we assign weights to the *county* level in such a way that minimizes the bias with respect to demographic variables of interest. After 10,000 simulations, we select the average weight for each county.
