# Supplementary material for: Characterizing collective physical distancing in the U.S. during the first nine months of the COVID-19 pandemic
Source: PLOS Digit Health. 2024 Feb 6;3(2):e0000430. doi: 10.1371/journal.pdig.0000430 (PMC10846712; doi:10.1371/journal.pdig.0000430)
Supplement: S5 Fig — (PDF) [file pdig.0000430.s010.pdf]

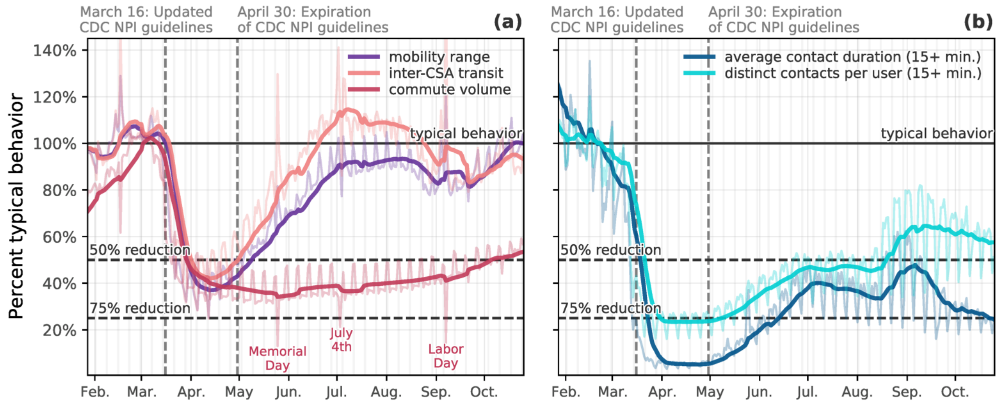

S5 Fig. Changes in mobility and person-to-person contacts over time (unweighted panel). Graphs show deviations from typical behavior for the same weekday, in the United States, using the *unweighted* panel. **(a)** Mobility: Individual mobility (radius of gyration), commute volume, and inter-CSA transit. **(b)** Contacts: Number of distinct contacts and average contact duration events outside of work and home. By the national declaration of emergency (March 13), reductions in spatial mobility measures had begun, reaching approximately 50% of typical values by April 1; while contact measures show a reduction greater than 75% by the same date. A 7-day rolling average is shown alongside each measure. Grey vertical lines denote weekends.
