# Supplementary material for: Characterizing collective physical distancing in the U.S. during the first nine months of the COVID-19 pandemic
Source: PLOS Digit Health. 2024 Feb 6;3(2):e0000430. doi: 10.1371/journal.pdig.0000430 (PMC10846712; doi:10.1371/journal.pdig.0000430)
Supplement: S6 Fig — (PDF) [file pdig.0000430.s011.pdf]

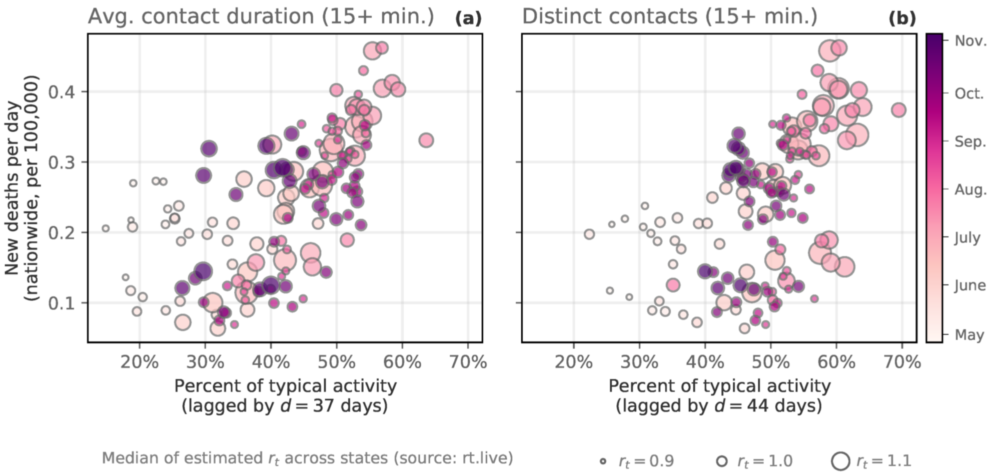

S6 Fig. Collective physical distancing and new deaths (unweighted panel). Replication of the analysis in Fig 5 using the unweighted panel to correlate daily contact measures nationwide with new reported deaths [30] between April 30 and November 5, 2020.
