# Supplementary material for: Characterizing collective physical distancing in the U.S. during the first nine months of the COVID-19 pandemic
Source: PLOS Digit Health. 2024 Feb 6;3(2):e0000430. doi: 10.1371/journal.pdig.0000430 (PMC10846712; doi:10.1371/journal.pdig.0000430)
Supplement: S7 Fig — (PDF) [file pdig.0000430.s012.pdf]

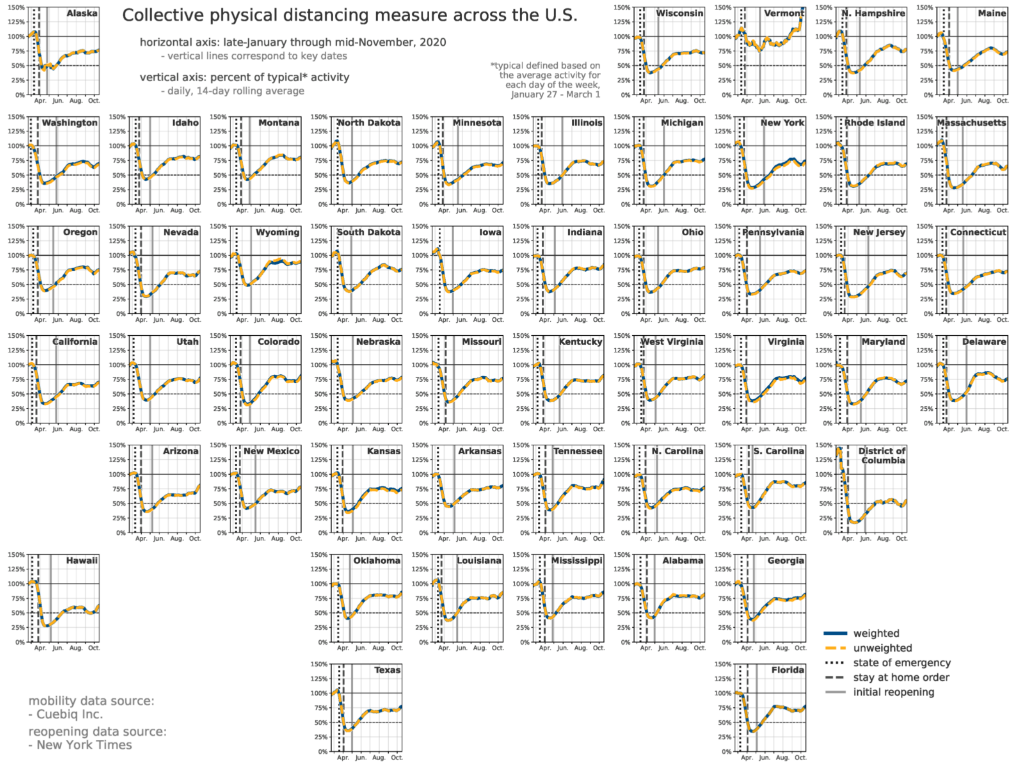

S7 Fig. Collective Physical Distancing: Weighted vs unweighted. For each state we report the aggregate measure of collective physical distancing, defined as the average of the typical daily commute volume, individual mobility range, inter-CSA transit, unique contacts outside of home and work, and total duration of contacts for the time frame listed, using the weighted panel (solid line) and the unweighted panel (dashed line).
