# Supplementary material for: Characterizing collective physical distancing in the U.S. during the first nine months of the COVID-19 pandemic
Source: PLOS Digit Health. 2024 Feb 6;3(2):e0000430. doi: 10.1371/journal.pdig.0000430 (PMC10846712; doi:10.1371/journal.pdig.0000430)
Supplement: S8 Fig — (PDF) [file pdig.0000430.s013.pdf]

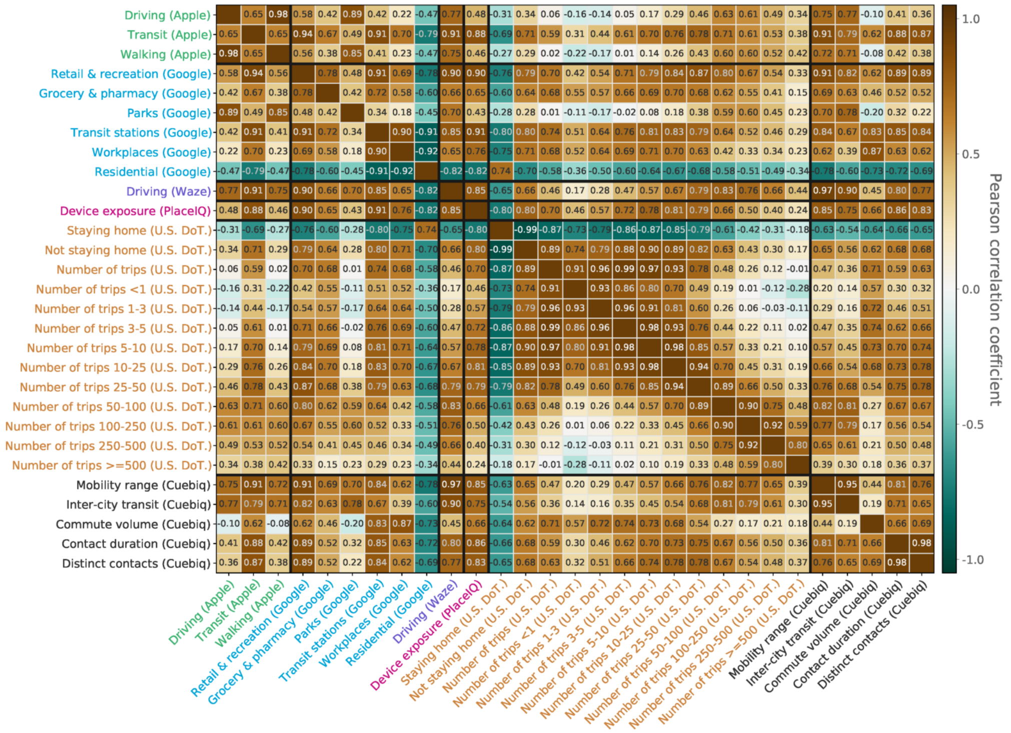

S8 Fig. Correlations across mobility datasets. Correlation matrix comparing the daily time series of mobility and contact patterns across different datasets.
