# Supplementary material for: Characterizing collective physical distancing in the U.S. during the first nine months of the COVID-19 pandemic
Source: PLOS Digit Health. 2024 Feb 6;3(2):e0000430. doi: 10.1371/journal.pdig.0000430 (PMC10846712; doi:10.1371/journal.pdig.0000430)
Supplement: S9 Fig — (PDF) [file pdig.0000430.s014.pdf]

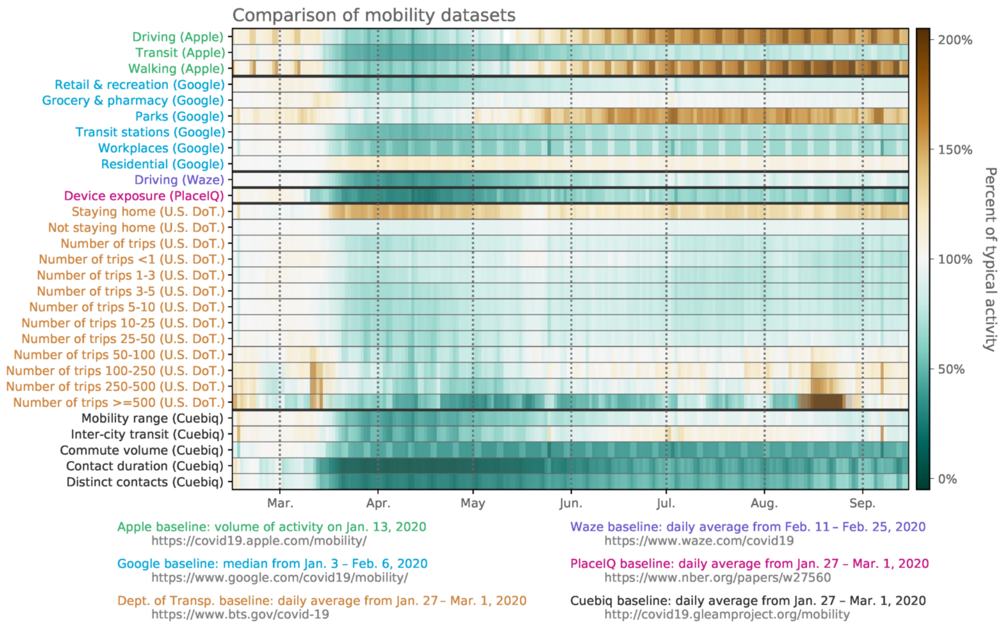

S9 Fig. Comparison across mobility datasets. While not meant to be a comprehensive comparison, this heatmap offers a coarse estimate of the similarities and differences of a variety of mobility measures from different datasets.
