# Supplementary material for: Characterizing collective physical distancing in the U.S. during the first nine months of the COVID-19 pandemic
Source: PLOS Digit Health. 2024 Feb 6;3(2):e0000430. doi: 10.1371/journal.pdig.0000430 (PMC10846712; doi:10.1371/journal.pdig.0000430)
Supplement: S10 Fig — (PDF) [file pdig.0000430.s015.pdf]

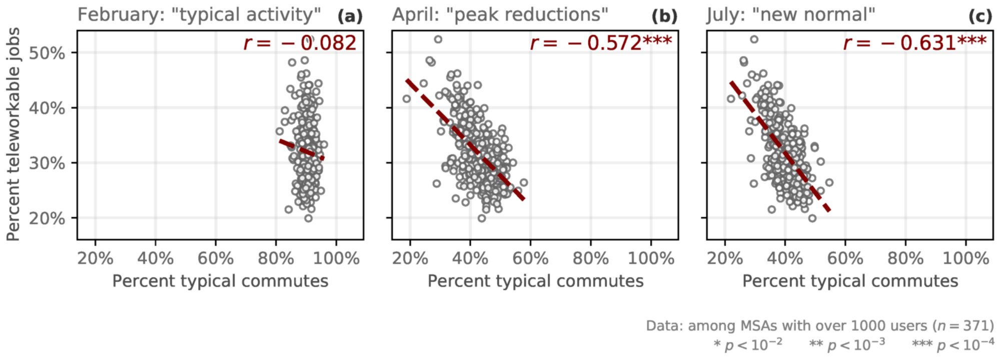

S10 Fig. Teleworking and commuting patterns. Grouping county-level employment data to the Metropolitan Statistical Area (MSA), we correlate commute volume with the percent of jobs that can readily transition to teleworking according to Dey et al. [29].
