# Supplementary material for: Characterizing collective physical distancing in the U.S. during the first nine months of the COVID-19 pandemic
Source: PLOS Digit Health. 2024 Feb 6;3(2):e0000430. doi: 10.1371/journal.pdig.0000430 (PMC10846712; doi:10.1371/journal.pdig.0000430)
Supplement: S11 Fig — (PDF) [file pdig.0000430.s016.pdf]

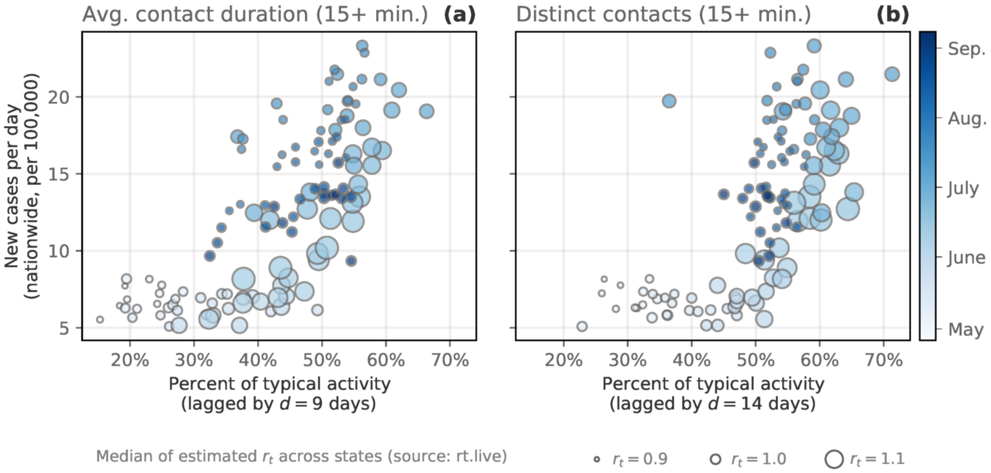

S11 Fig. Collective physical distancing and new infections. Correlating contact measures nationwide (lagged) with new reported cases daily per 100,000 (data from *The COVID Tracking Project* [30]) between April 30 and September 5, 2020. A lag of d days was selected for each state so as to maximize the R-squared of the correlation between average contact duration and new infections. **(a)** Average contact duration **(b)** Distinct contacts. In each subplot, darker colors indicate later dates and marker size corresponds to an estimate of the median R_t across all 50 states and District of Columbia (source: `rt.live`).
