# Supplementary material for: Characterizing collective physical distancing in the U.S. during the first nine months of the COVID-19 pandemic
Source: PLOS Digit Health. 2024 Feb 6;3(2):e0000430. doi: 10.1371/journal.pdig.0000430 (PMC10846712; doi:10.1371/journal.pdig.0000430)
Supplement: S12 Fig — (PDF) [file pdig.0000430.s017.pdf]

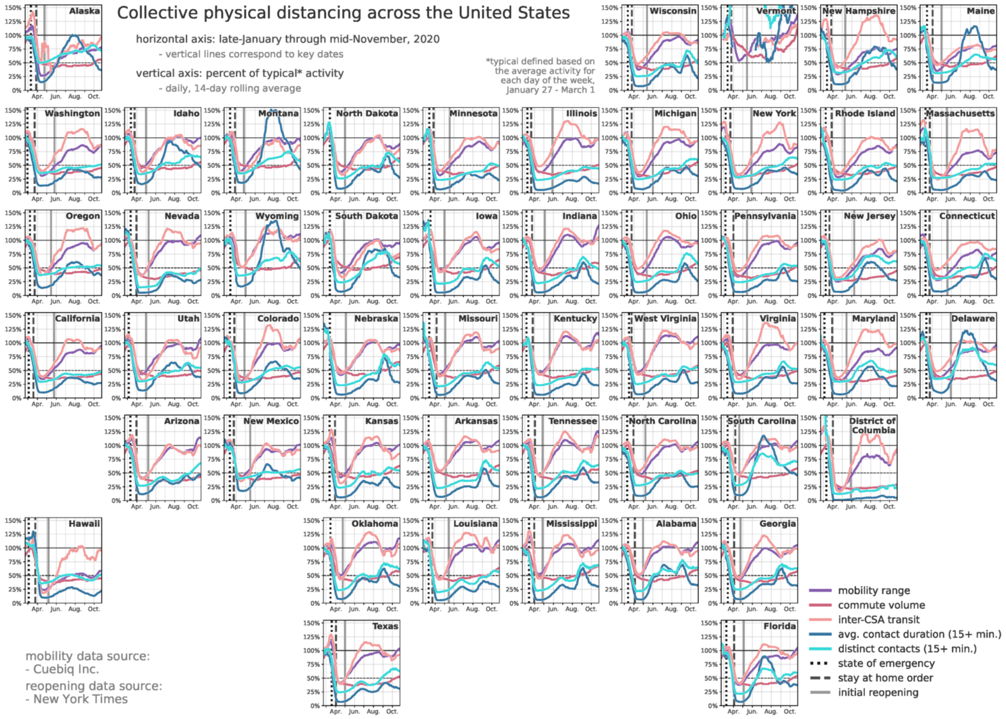

S12 Fig. Collective physical distancing across every state. Grid cartogram including the five measures shown in Fig 1, for all 50 states and District of Columbia.
