# Supplementary material for: Characterizing collective physical distancing in the U.S. during the first nine months of the COVID-19 pandemic
Source: PLOS Digit Health. 2024 Feb 6;3(2):e0000430. doi: 10.1371/journal.pdig.0000430 (PMC10846712; doi:10.1371/journal.pdig.0000430)
Supplement: S14 Fig — (PDF) [file pdig.0000430.s019.pdf]

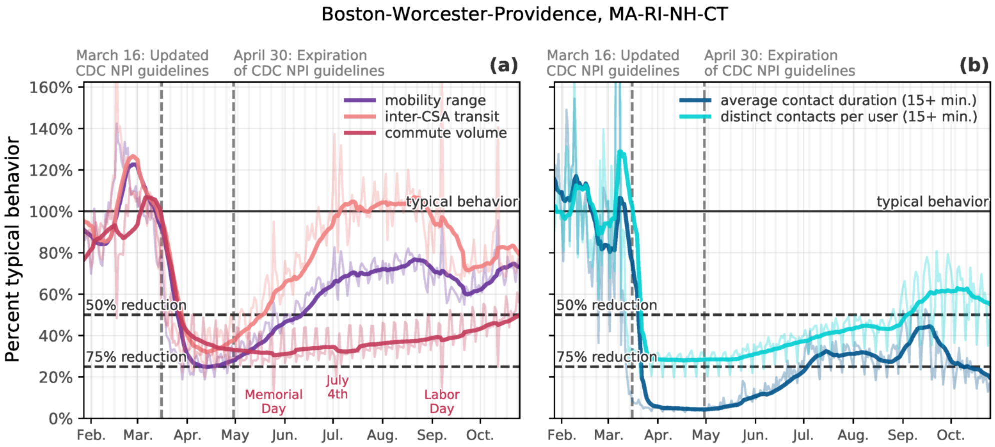

S14 Fig. Changes in mobility and person-to-person contacts over time in Boston-Worcester-Providence, MA-RI-NH-CT. Graphs show deviations from typical behavior for the same weekday.
