# Supplementary material for: Characterizing collective physical distancing in the U.S. during the first nine months of the COVID-19 pandemic
Source: PLOS Digit Health. 2024 Feb 6;3(2):e0000430. doi: 10.1371/journal.pdig.0000430 (PMC10846712; doi:10.1371/journal.pdig.0000430)
Supplement: S15 Fig — (PDF) [file pdig.0000430.s020.pdf]

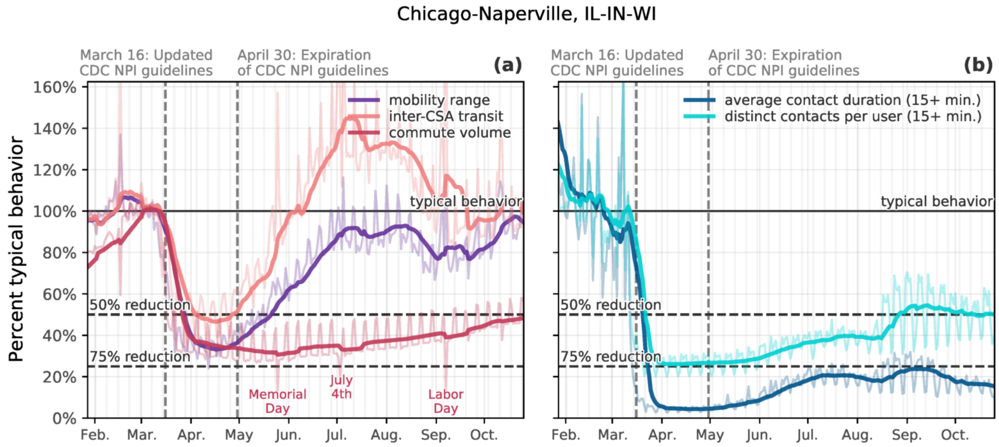

S15 Fig. Changes in mobility and person-to-person contacts over time in Chicago-Naperville, IL-IN-WI. Graphs show deviations from typical behavior for the same weekday.
